# Supplementary material for: Fast viral dynamics revealed by microsecond time-resolved cryo-EM
Source: Nat Commun. 2023 Sep 13;14:5649. doi: 10.1038/s41467-023-41444-x (PMC10499870; doi:10.1038/s41467-023-41444-x)
Supplement: Supplementary file 1 — Supplementary Information [file 41467_2023_41444_MOESM1_ESM.pdf]

Supplementary Information

Fast Viral Dynamics Revealed by Microsecond Time-Resolved Cryo-EM

Oliver F. Harder†, Sarah V. Barrass†, Marcel Drabbels, and Ulrich J. Lorenz*

Affiliation: Ecole Polytechnique Fédérale de Lausanne (EPFL), Laboratory of Molecular Nanodynamics, CH-1015 Lausanne, Switzerland

* Corresponding author. Email: ulrich.lorenz@epfl.ch

† These authors contributed equally: Oliver F. Harder, Sarah V. Barrass

Supplementary Methods

A. Sample preparation

Cowpea chlorotic mottle virus (CCMV) was provided by the group of Prof. Jeroen Cornelissen at the University of Twente. Cryo samples of contracted CCMV (pH 5, free of divalent ions were prepared by applying 3 µl of the sample solution (11.5 mg/ml in 100 mM sodium acetate buffer, 1 mM sodium azide, 1 mM EDTA) to glow discharged UltrAuFoil grids (R1.2/1.3, 300 mesh, Quantifoil), which were plunge frozen with a Thermo Fisher Vitrobot Mark IV (10 °C, 95 % relative humidity, 3 s blotting time, blotting force of 10). Cryo samples of extended CCMV (pH 7.6) were prepared from the sample solution through buffer exchange with a centrifugal concentrator (MWCO 50 kDa, 10°C). The sample solution (23 mg/ml in 10 mM Tris buffer, 50 mM sodium chloride, 1mM EDTA) was then plunge frozen under the same conditions. For pH jump experiments, NPE-caged-proton (Bio-Techne, 120 mM) was added to this solution in a 1:6 volume ratio prior to plunge freezing.

In order to release the photoacid, cryo samples were placed in a Linkam cryo stage and irradiated with a nanosecond pulsed UV laser for 30 min (Bright Solutions Wedge, 266 nm, 35 mW, 7.1±0.1 mm FWHM spot size in the sample plane, as determined from a knife edge scan). The pH of the sample after release of the photoacid was determined as follows. A frozen droplet of the same sample solution (3 µl) was UV irradiated under identical conditions, after which the droplet was melted. A measurement with a pH strip yielded a pH of 4.5± 0.5.

In order to determine that UV irradiation does not cause contraction of the particles in the absence of a photoacid (Supplementary Fig. 2), cryo samples of extended CCMV (without photoacid) were irradiated under identical conditions.

B. In-situ revitrification

Revitrification experiments were performed *in situ* with a modified JEOL 2200FS transmission electron microscope [1] as previously described [2]. Microsecond laser pulses for revitrification (532 nm wavelength,

about 100 mW power) were obtained by chopping the output of a continuous laser (Laser Quantum, Ventus 532) with an acousto-optic modulator (AA Opto-Electronic). The laser beam was focused to a spot size of 28±2 µm FWHM in the sample plane, as determined from a knife edge scan. Sample areas were melted and revitrified with the laser beam centered onto a grid square. We estimate that both the melting and the revitrification process occur on a timescale of about 5-7 µs, as determined from heat transfer simulations, which we previously found to describe the experiment well [2]. Evaporation of the sample in the vacuum of the electron microscope reduces its thickness by several tens of nanometers, with the change in thickness depending on the exact sample thickness and plateau temperature reached. This number is likely smaller for the actual cryo samples used in our experiment, which contain buffer, photoacid, and proteins, all of which likely reduce the evaporation coefficient of the solution.

C. Data collection

Data were collected at the Dubochet Center for Imaging in Lausanne using a Titan Krios microscope equipped with Falcon IV detector and a SelectrisX energy filter. The data acquisition parameters for the different data sets are listed in supplementary table 1.

D. Single-particle reconstructions

Single-particle reconstructions were performed in CryoSPARC 4.0.1 [3]. The following workflow was used, with details for the different datasets are provided in Supplementary Figs. 1 and 2. The micrographs were patch motion corrected, and movies with a total full-frame motion distance of more than 100 pixels were discarded. Contrast transfer function (CTF) estimation was performed using Patch CTF in CryoSPARC. Micrographs with an estimated resolution of less than 10 Å or an astigmatism of over 2000 Å were discarded, as well as micrographs containing hexagonal or cubic ice. Particles were picked using manual picking or blob picking, followed by template picking and were extracted with a box size of 768 pixels. Following 2D classification and *ab initio* reconstruction, the best classes resulting from heterogeneous refinement (Icosahedral symmetry) were further refined using homogeneous or non-uniform refinement with Icosahedral symmetry imposed. Additional refinement steps included CTF refinement, per particle defocus refinement, and Ewald sphere correction. The global and local resolution was estimated using an gold-

standard FSC threshold of 0.143. The structures were visualized with UCSF ChimeraX 1.5 [4,5]. The reconstructions colored by local resolution, and the angular distribution of particles used in the final reconstructions are shown in Supplementary Fig. 3a–e and 3f–j, respectively.

E. Variability analysis

The variability analysis shown in Fig. 3e was performed on a combined dataset containing 21,675 refined particles each of the extended, partially contracted, and fully contracted CCMV. Three variability components and a low-pass filter resolution of 10 Å were used. In Fig. 3e, the particle distribution is shown as a function of the first two components. To analyze the motions of the capsid proteins during contraction, the particle distribution was divided into 30 equal slices along the first component, and each slice was homogeneously reconstructed with icosahedral symmetry imposed.

F. Model building

A model of contracted CCMV capsid was obtained with the following procedure. The model of contracted CCMV from PDB:1ZA7 [6] was protonated using the APBS-PDB2PQR software suite [7] with the pH set to 5 and rigidly fit into the auto-sharpened contracted CCMV map using UCSF ChimeraX 1.4 [4,5]. The asymmetric unit was then iteratively refined using the real space refine tool in PHENIX 1.20.1-4487 [8], with secondary structure and non-crystallographic symmetry constraints imposed, and the ISOLDE plugin [9] in UCSF ChimeraX. The refinement was finalized using a map section containing the asymmetric unit as well as the chains in its proximity.

A model of the extended CCMV capsid was obtained with a similar procedure. The model of the contracted form obtained above was set to the protonation state at pH 7.6 and rigidly fit into the auto-sharpened map of extended CCMV. The asymmetric unit was then refined as above while imposing torsional and adaptive distance restraints derived from the contracted structure. Imposing these restraints helped prevent unphysical geometries from occurring during the refinement in the 3.9 Å map. The quality of the models were evaluated using MolProbity [10] and PHENIX [8]. The atomic models of the asymmetric units are shown in Supplementary Fig 5.

G. Analysis of the motions of the capsid proteins during contraction

In order to analyze the motions of the capsid proteins during contraction, the particle distribution in Fig. 3e was divided into 30 slices along the first component, and a reconstruction was obtained for each slice. The model of the extended state was then rigidly fit into the reconstructions of slices 1–12 (extended and partially contracted configurations), and the model of the contracted state into the reconstructions of slices 25–30 (contracted configurations). The positions of the alpha carbons were then used to extract the geometric parameters reported in Fig. 4.

The capsid diameter (Fig. 4b) was measured along 5-fold symmetry axis and corresponds to the largest distance of two alpha carbons along this direction. The angles of the rotation of the pentamers around the 5-fold axis (Fig. 4b) were determined from the displacements of the centers of mass of the B proteins. The rotation angles of the hexamers around the 3-fold axis (Fig. 4c) represent an average of the rotation angles of the A and C proteins, which were calculated in the same manner. After subtracting the rotation of the capsomers, the superimposed rotation of each capsid protein around its center for mass was then calculated (Fig. 4d).

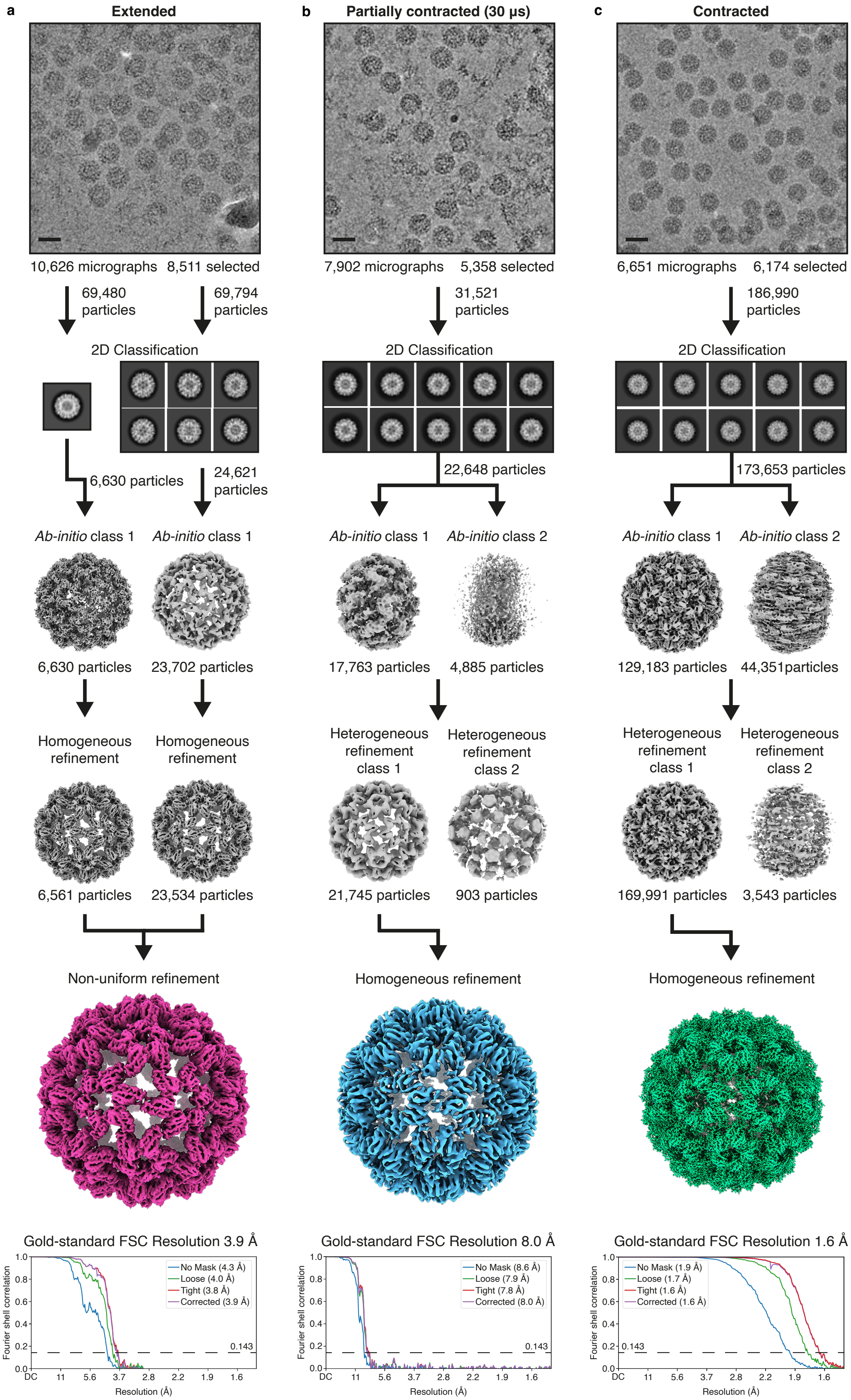

a
Extended
10,626 micrographs
8,511 selected
69,480 particles
69,794 particles
2D Classification
6,630 particles
24,621 particles
Ab-initio class 1
Ab-initio class 1
6,630 particles
23,702 particles
Homogeneous refinement
Homogeneous refinement
6,561 particles
23,534 particles
Non-uniform refinement
Gold-standard FSC Resolution 3.9 Å
No Mask (4.3 Å)
Loose (4.0 Å)
Tight (3.8 Å)
Corrected (3.9 Å)
0.143
Fourier shell correlation
Resolution (Å)
b
Partially contracted (30 μs)
7,902 micrographs
5,358 selected
31,521 particles
2D Classification
22,648 particles
Ab-initio class 1
Ab-initio class 2
17,763 particles
4,885 particles
Heterogeneous refinement class 1
Heterogeneous refinement class 2
21,745 particles
903 particles
Homogeneous refinement
Gold-standard FSC Resolution 8.0 Å
No Mask (8.6 Å)
Loose (7.9 Å)
Tight (7.8 Å)
Corrected (8.0 Å)
0.143
Fourier shell correlation
Resolution (Å)
c
Contracted
6,651 micrographs
6,174 selected
186,990 particles
2D Classification
173,653 particles
Ab-initio class 1
Ab-initio class 2
129,183 particles
44,351particles
Heterogeneous refinement class 1
Heterogeneous refinement class 2
169,991 particles
3,543 particles
Homogeneous refinement
Gold-standard FSC Resolution 1.6 Å
No Mask (1.9 Å)
Loose (1.7 Å)
Tight (1.6 Å)
Corrected (1.6 Å)
0.143
Fourier shell correlation
Resolution (Å)

Supplementary Fig. 1 | Single-particle reconstruction workflows for the extended (a), partially contracted (b), and fully contracted configurations of the CCMV capsid (c). Gold Standard Fourier Shell Correlations are shown for each data set, with the dashed black line indicating the 0.143 cutoff. For the extended configuration in (**a**), two datasets were processed separately and merged in the final reconstruction. Note that the micrograph in (**b**) shows some partially disassembled particles. These particles have likely already disassembled during the preparation of the extended state of CCMV, which is unstable and prone to disassembly. Melting and revitrification does not seem to noticeably increase the number of such partially disassembled particles (see also the micrographs in Supplementary Fig. 2a and 2b), although we cannot exclude that protonation of partially disassembled configurations leads to the further disassembly during laser melting.

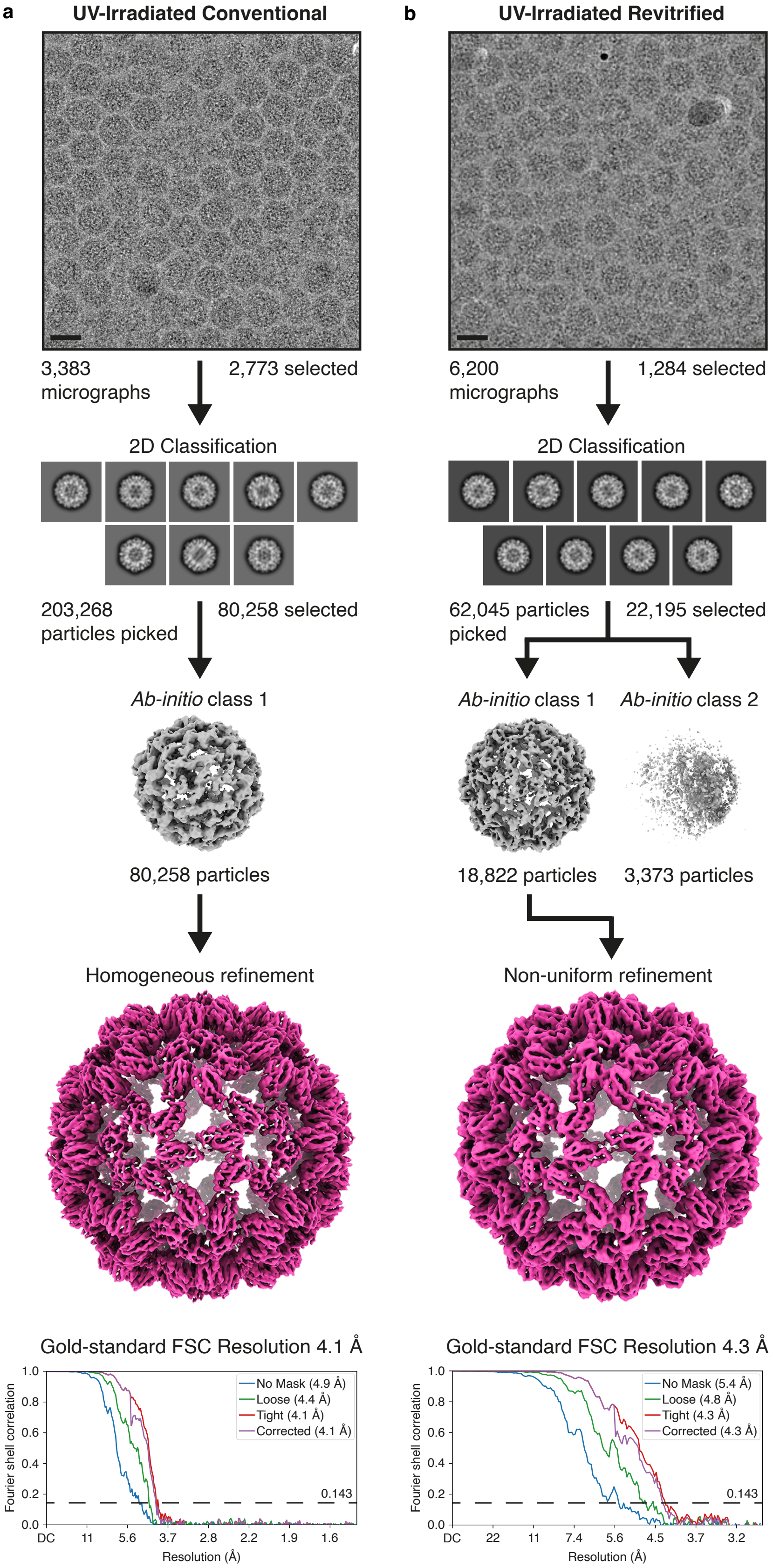
a
UV-Irradiated Conventional
3,383 micrographs
2,773 selected
2D Classification
203,268 particles picked
80,258 selected
Ab-initio class 1
80,258 particles
Homogeneous refinement
Gold-standard FSC Resolution 4.1 Å
No Mask (4.9 Å)
Loose (4.4 Å)
Tight (4.1 Å)
Corrected (4.1 Å)
0.143
Fourier shell correlation
Resolution (Å)
b
UV-Irradiated Revitrified
6,200 micrographs
1,284 selected
2D Classification
62,045 particles picked
22,195 selected
Ab-initio class 1
Ab-initio class 2
18,822 particles
3,373 particles
Non-uniform refinement
Gold-standard FSC Resolution 4.3 Å
No Mask (5.4 Å)
Loose (4.8 Å)
Tight (4.3 Å)
Corrected (4.3 Å)
0.143
Fourier shell correlation
Resolution (Å)

Supplementary Fig. 2 | Control experiment demonstrating that the contraction of CCMV is not induced by any effect that UV irradiation might have on the particles, but instead by the pH jump created through the release of the photoacid. A sample of the extended configuration of CCMV was prepared at pH 7.5 in the absence of any photoacid and irradiated with UV light, using the same fluence as in the experiment of Fig. 3. Single-particle reconstruction workflows for conventional and revitrified sample areas are shown in (**a**) and (**b**), respectively. The reconstructions reveal that melting and revitrification does not cause the particles to contract. This demonstrates that UV irradiation alone does not induce contraction. Gold Standard Fourier Shell Correlations are shown for each data set, with the dashed black line indicating the 0.143 cutoff.

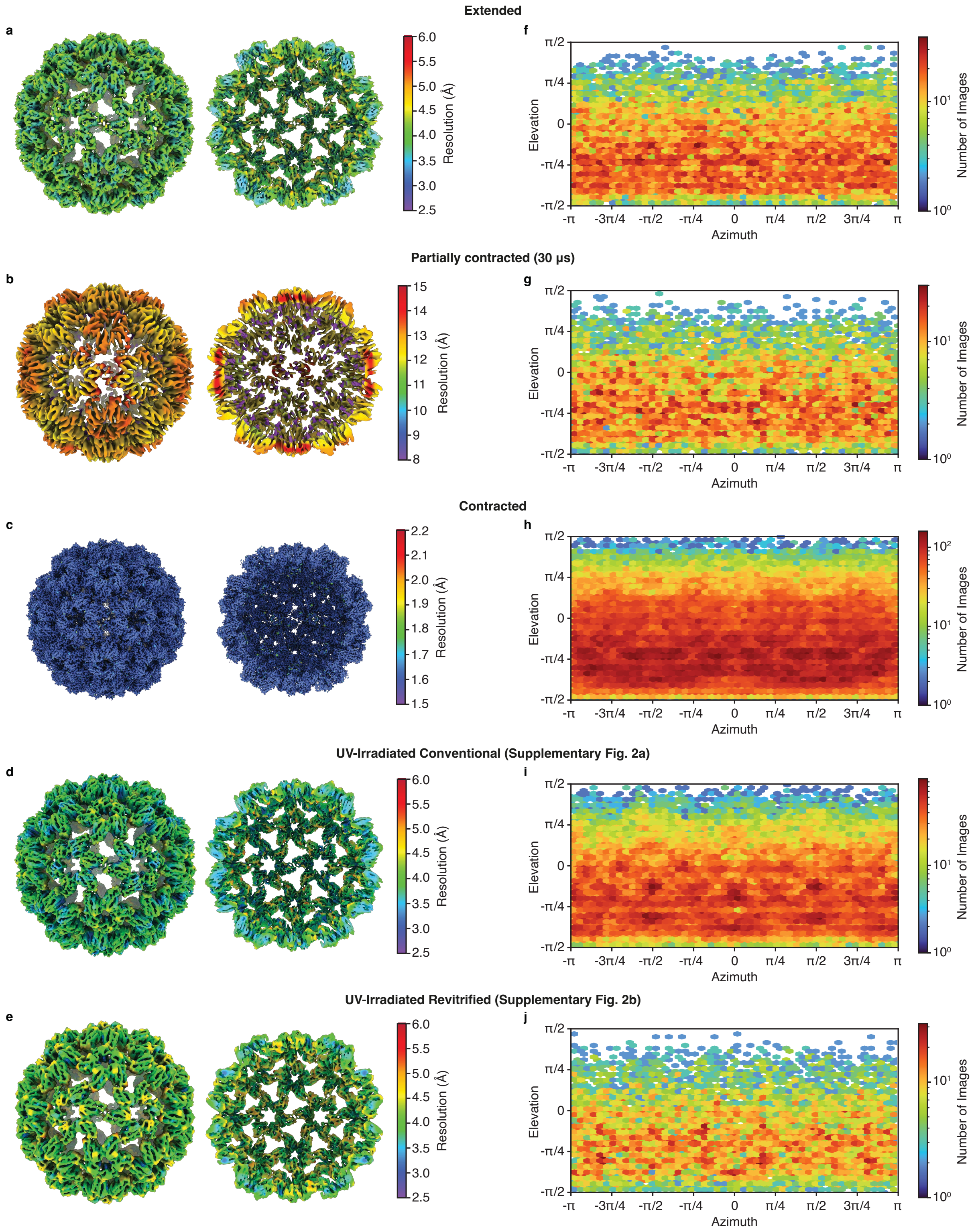

Supplementary Fig. 3 | Local resolution, and angular distribution for the reconstructions shown in the manuscript. a, extended; **b**, partially contracted; **c**, contracted; **d-e** UV-irradiated**.**

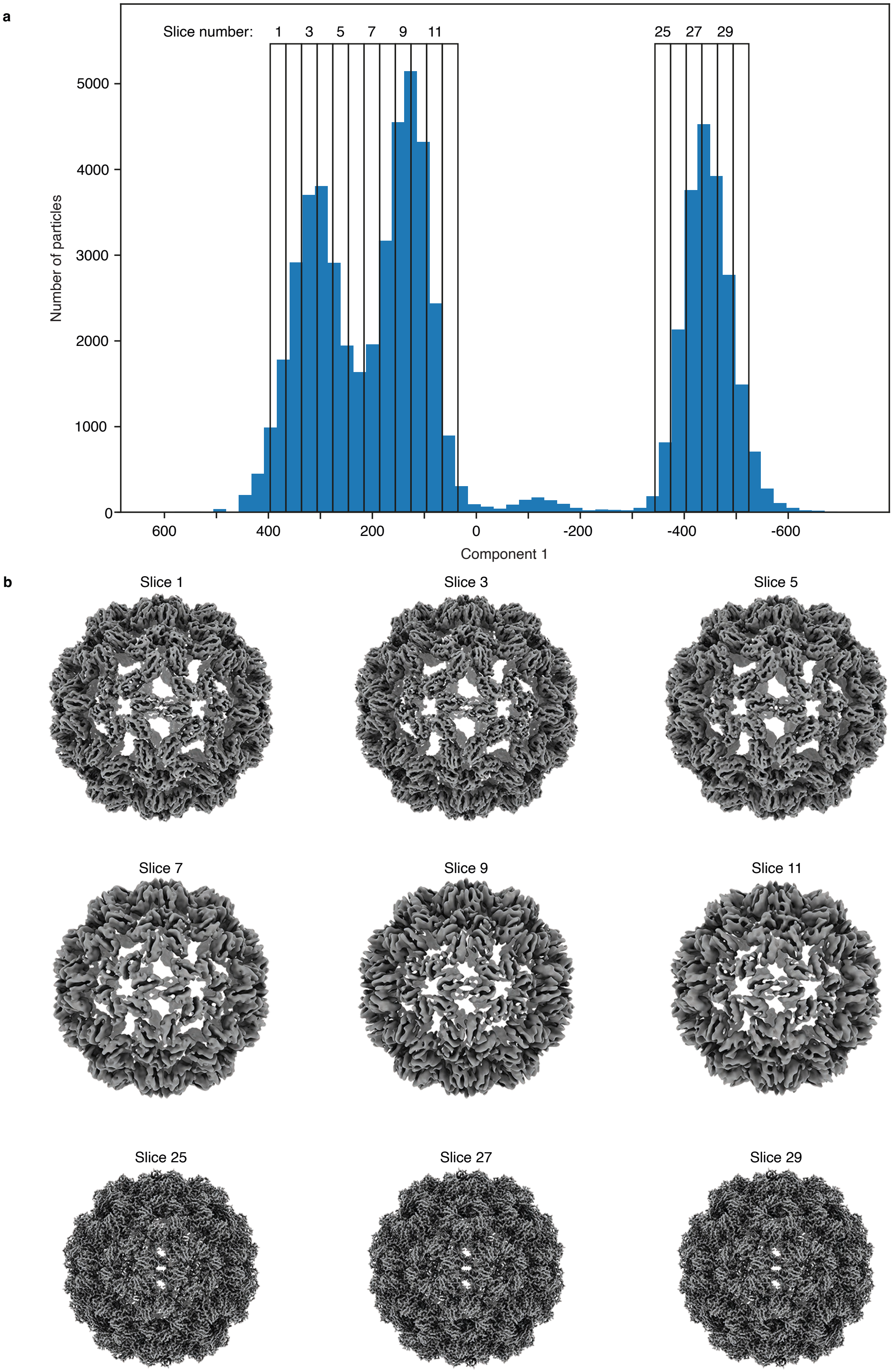
a
Slice number: 1 3 5 7 9 11 25 27 29
Number of particles
0
1000
2000
3000
4000
5000
600
400
200
0
-200
-400
-600
Component 1
b
Slice 1
Slice 3
Slice 5
Slice 7
Slice 9
Slice 11
Slice 25
Slice 27
Slice 29

Supplementary Fig. 4 | Variability analysis of a combined dataset of the extended, partially contracted, and fully contracted configurations of CCMV from Fig. 3e. a, The particle distribution from Fig. 3e is shown as a function of the first variability component. The distribution is divided into 30 slices along the first component. **b**, A reconstruction is performed for the particles in each slice, which is used to determine the motions of the capsid proteins (Fig. 4). Here, reconstructions are shown for representative slices, which illustrate the pH jump induced contraction process.

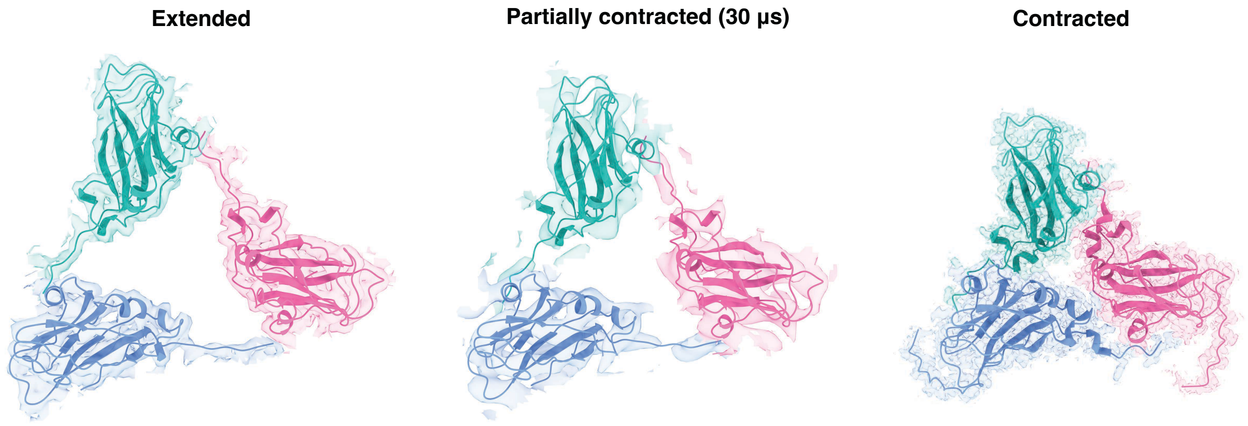

Supplementary Fig. 5 | Models of the asymmetric unit of the extended, partially contracted, and fully contracted states.

Supplementary Table 1 | Data collection parameters, and reconstruction and modelling statistics.

Figure<br>**Name**	Fig. 3c<br>**Contracted**	Fig. 3b<br>**Partially contracted (30 µs)**	Fig. 3a<br>**Extended**	Sup. Fig. 2b<br>**UV-irradiated Revitrified**	Sup. Fig. 2a<br>**UV-irradiate Conventional**
EMDB accession	(EMDB-16400)	(EMDB-16798)	(EMDB-16790)	(EMDB-16858)	(EMDB-16857)
PDB accession	(PDB 8C38)		(PDB 8CPY)		
**Data collection**					
Microscope	Thermo Fisher Titan Krios				
Detector	Falcon 4				
Energy Filter	SelectrisX, 10 eV band width				
Magnification	165,000				
Voltage (kV)	300				
Electron exposure (e^{-}/Å^2)	50				
Defocus range (µm)	-0.3 – -0.9				
Pixel size (Å)	0.726				
**Data processing**					
Initial movies (no.)	6,651	7,902	10,626	3,383	6,200
Symmetry imposed	Icosahedral (I)	Icosahedral (I)	Icosahedral (I)	Icosahedral (I)	Icosahedral (I)
Initial particle images (no.)	186,990	31,521	139,274	62,045	203,268
Final particle images (no.)	169,835	21,675	30,095	18,822	79,910
Map resolution (Å)	1.6	3.9	8.0	4.3	4.1
FSC threshold	0.143	0.143	0.143	0.143	0.143
Map resolution range (Å)	1.63 – 2.16	6.76 – 9.84	3.41 – 15.06	3.17 – 6.48	2.86 – 6.35
**Refinement**					
Initial model used (PDB code)	1ZA7	-	8C38	-	-
Model resolution (Å)	1.61	-	3.81	-	-
FSC threshold	0.143	-	0.143	-	-
Model resolution range (Å)	1.61 – 1.65	-	3.81 – 3.93	-	-
Map sharpening *B* factor (Å^2)	40.7	1017.9	133.1	143.9	153.5
Model composition					
Non-hydrogen atoms	3,593	-	3,307	-	-
Protein residues	479	-	441	-	-
Average *B* factors (Å^2)					
Protein	4.17	-	92.09	-	-
R.m.s. deviations					
Bond lengths (Å)	0.003	-	0.005	-	-
Bond angles (°)	0.553	-	1.000	-	-
Validation					
MolProbity score	0.87	-	1.67	-	-
Clashscore	1.38	-	8.12	-	-
Poor rotamers (%)	0.00	-	0.28	-	-
Ramachandran plot					
Favored (%)	98.73	-	96.55	-	-
Allowed (%)	1.27	-	3.45	-	-
Disallowed (%)	0.00	-	0.00	-	-

Supplementary References

1. Olshin, P. K., Voss, J. M., Drabbels, M. & Lorenz, U. J. Real-time observation of jumping and spinning nanodroplets. *Struct. Dyn.* **7**, 011101 (2020).
2. Bongiovanni, G., Harder, O. F., Voss, J. M., Drabbels, M. & Lorenz, U. J. Near-atomic resolution reconstructions from *in situ* revitrified cryo samples. *Acta Crystallogr. Sect. Struct. Biol.* **79**, 473–478 (2023).
3. Punjani, A., Rubinstein, J. L., Fleet, D. J. & Brubaker, M. A. cryoSPARC: algorithms for rapid unsupervised cryo-EM structure determination. *Nat. Methods* **14**, 290–296 (2017).
4. Pettersen, E. F. *et al.* UCSF CHIMERAX : Structure visualization for researchers, educators, and developers. *Protein Sci.* **30**, 70–82 (2021).
5. Goddard, T. D. *et al.* UCSF ChimeraX: Meeting modern challenges in visualization and analysis: UCSF ChimeraX Visualization System. *Protein Sci.* **27**, 14–25 (2018).
6. Speir, J. A. *et al.* Enhanced Local Symmetry Interactions Globally Stabilize a Mutant Virus Capsid That Maintains Infectivity and Capsid Dynamics. *J. Virol.* **80**, 3582–3591 (2006).
7. Jurrus, E. *et al.* Improvements to the APBS biomolecular solvation software suite. *Protein Sci.* **27**, 112–128 (2018).
8. Liebschner, D. *et al.* Macromolecular structure determination using X-rays, neutrons and electrons: recent developments in *Phenix*. *Acta Crystallogr. Sect. Struct. Biol.* **75**, 861–877 (2019).
9. Croll, T. I. *ISOLDE* : a physically realistic environment for model building into low-resolution electron-density maps. *Acta Crystallogr. Sect. Struct. Biol.* **74**, 519–530 (2018).
10. Williams, C. J. *et al.* MolProbity: More and better reference data for improved all-atom structure validation: PROTEIN SCIENCE.ORG. *Protein Sci.* **27**, 293–315 (2018).
